# Ligand-induced rapid skeletal muscle atrophy in HSA-Fv2E-PERK transgenic mice

**DOI:** 10.1371/journal.pone.0179955

**Published:** 2017-06-23

**Authors:** Masato Miyake, Masashi Kuroda, Hiroshi Kiyonari, Kenji Takehana, Satoshi Hisanaga, Masatoshi Morimoto, Jun Zhang, Miho Oyadomari, Hiroshi Sakaue, Seiichi Oyadomari

**Affiliations:** 1 Division of Molecular Biology, Institute for Genome Research, Institute of Advanced Medical Sciences, Tokushima University, Tokushima, Japan; 2 Department of Molecular Research, Diabetes Therapeutics and Research Center, Tokushima University, Tokushima, Japan; 3 Fujii Memorial Institute of Medical Sciences, Tokushima University, Tokushima, Japan; 4 Department of Nutrition and Metabolism, Institute of Biomedical Sciences, Tokushima University Graduate School, Tokushima, Japan; 5 Animal Resource Development Unit and Genetic Engineering Team, RIKEN Center for Life Science Technologies, Kobe, Japan; 6 Department of R&D planning, EA Pharma Co.,Ltd., Tokyo, Japan; University of Louisville School of Medicine, UNITED STATES

## Abstract

**Background:**

Formation of 43S and 48S preinitiation complexes plays an important role in muscle protein synthesis. There is no muscle-wasting mouse model caused by a repressed 43S preinitiation complex assembly.

**Objective:**

The aim of the present study was to develop a convenient mouse model of skeletal muscle wasting with repressed 43S preinitiation complex assembly.

**Material and methods:**

A ligand-activatable PERK derivative Fv2E-PERK causes the phosphorylation of eukaryotic initiation factor 2α (eIF2α), which inhibits 43S preinitiation complex assembly. Thus, muscle atrophic phenotypes, intracellular signaling pathways, and intracellular free amino acid profiles were investigated in human skeletal muscle α-actin (HSA) promoter-driven Fv2E-PERK transgenic (Tg) mice.

**Results:**

HSA-Fv2E-PERK Tg mice treated with the artificial dimerizer AP20187 phosphorylates eIF2α in skeletal muscles and leads to severe muscle atrophy within a few days of ligand injection. Muscle atrophy was accompanied by a counter regulatory activation of mTORC1 signaling. Moreover, intracellular free amino acid levels were distinctively altered in the skeletal muscles of HSA-Fv2E-PERK Tg mice.

**Conclusions:**

As a novel model of muscle wasting, HSA-Fv2E-PERK Tg mice provide a convenient tool for studying the pathogenesis of muscle loss and for assessing putative therapeutics.

## Introduction

Skeletal muscles are involved in fundamental functions, such as enabling locomotion, maintaining body temperature, and storing nutrients. Muscle mass is determined by a balance between anabolic and catabolic processes. Skeletal muscle wasting occurs in several disorders, including sarcopenia, infection, cancer cachexia, disuse and denervated muscle [1, 2]. Muscle wasting results from an imbalance between protein degradation and protein synthesis [3].

Protein degradation is controlled by three proteolytic pathways, i.e., the ubiquitin–proteasome, autophagy–lysosome, and calcium-dependent calpain systems. Calpain is suggested to be the early rate-limiting factor in myofibrillar proteolysis during muscle wasting [1]. They can degrade proteins required for the assembly and scaffolding of myofibrillar proteins, such as desmin, filamen, C-protein, tropomyosin, troponin T, troponin I, titin, nebulin, vimentin, gelsolin, and vinculin [4]. In addition, ubiquitin–proteasome systems degrade the bulk of myofibrillar proteins during muscle wasting [5, 6]. Recent studies using the genetic manipulation of autophagy regulators revealed that a fine equilibrium of autophagic flux is necessary to preserve muscle mass [7]. Excessive autophagy causes an acute muscle loss owing to the imprudent clearance of necessary cellular components. Insufficient autophagy causes a chronic muscle loss due to the accumulation of damaged or aged cellular components. Thus, there is increasing evidence that increased protein degradation due to the dysregulation of these proteolytic pathways contributes to muscle wasting.

Although protein degradation is an important contributor to the development and progression of muscle wasting, an impairment of protein synthesis is also known to be involved in muscle wasting under atrophic conditions, such as sepsis, sarcopenia, and cancer cachexia [3]. The process of protein synthesis can be divided into initiation, elongation, termination, and recycling [8]. The initiation step plays a major role in modulating the protein synthesis rate [9]. One of the rate-limiting steps in initiation is the recruitment of mRNA to the 43S preinitiation complex by the eIF4E–eIF4G–eIF4A cap-binding complex, resulting in a complex called the 48S preinitiation complex. Signaling through the mammalian target of rapamycin complex 1 (mTORC1) plays a key role in this process. mTORC1 phosphorylates 4EBP1 allowing cap-dependent protein synthesis, given that unphosphorylated 4EBP1 binds to the mRNA cap and prevents the cap-dependent mRNA binding to the 43S preinitiation complex. In addition to 4EBP1, mTORC1 also phosphorylates S6K, which is subsequently phosphorylates eIF4B, an eIF4A helicase activator that is activated by phosphorylation, and PDCD4, an eIF4A inhibitor that is inhibited by phosphorylation. Similar to the function of phosphorylated 4EBP1 to enhance cap-dependent protein synthesis, PDCD and eIF4B phosphorylation causes the release of eIF4A to interact with eIF4G and the assembly of eIF4A into the eIF4E–eIF4G–eIF4A cap-binding complex.

Another rate-limiting step of initiation is the formation of a ternary complex comprising eIF2, GTP, and initiator methionyl-tRNA (Met-tRNAi), which subsequently binds to the 40S ribosomal subunit to form the 43S preinitiation complex [8]. The phosphorylation of the eIF2 α-subunit on Ser51 is a critical regulator in this process. Phosphorylated eIF2α binds and inhibits the guanine nucleotide exchange factor eIF2B, which is required for recycling the GDP-bound eIF2 into the translationally active GTP-bound form. As the GTP-bound eIF2 is hydrolyzed at a late initiation step, eIF2B activity inhibition results in the accumulation of the GDP-bound eIF2 that has limited affinity for Met-tRNAi, leading to a decrease in protein synthesis. The eIF2α phosphorylation is regulated by four eIF2α kinases, such as general control nonderepressible 2 (GCN2), double-stranded RNA-dependent protein kinase (PKR), PKR-like endoplasmic reticulum-associated protein kinase (PERK) and heme-regulated inhibitor (HRI), and eIF2α phosphatases, containing CReP and GADD34 [10], all of which are activated by distinct cellular stresses, such as amino acid deficiency for GCN2, RNA virus infection for PKR, endoplasmic reticulum stress for PERK, and heme depletion for HRI. The eIF2α dephosphorylation is regulated by constitutively expressed CReP and stress-induced GADD34.

In the previous study, we explored the role of eIF2α phosphorylation in skeletal muscles and showed that eIF2α was phosphorylated by exercise and cold exposure in skeletal muscles and that mild eIF2α phosphorylation caused transcriptional induction to regulate energy consumption [11]. Moreover, we also found that strong eIF2α phosphorylation suppressed muscle protein synthesis [11]. Here, we further investigated the previously established transgenic mice harboring a ligand-activated skeletal muscle-specific eIF2α phosphorylation. In the present study, we showed that strong and constitutive eIF2α phosphorylation in skeletal muscles induces rapid muscle wasting, offering a convenient mouse model of rapid-onset muscle wasting with reduced muscle protein synthesis. This model may facilitate the development of novel therapies for muscle wasting.

## Materials and methods

### Mice

HSA-Fv2E-PERK Tg mice (Accession No. CDB0514T: http://www2.clst.riken.jp/arg/TG%20mutant%20mice%20list.html) were generated on a C57Bl/6N background and maintained as described previously [11]. Briefly, a 4.9-kb fragment containing the human skeletal muscle α-actin promoter (2.2 kb), the Fv2E regulatory domain (0.7 kb), the mouse PERK kinase domain (1.7 kb), and the bovine growth hormone poly(A) sequence (0.3 kb) was constructed and expressed in mouse skeletal muscles. Mice were genotyped using polymerase chain reaction (PCR) with primers for the Fv2E domain (Table 1). AP21087 (AP; Apexbio) or vehicle (4% ethanol, 10% polyethylene glycol-400, and 1.75% Tween-20 in water) were injected intraperitoneally once a day for 7 days. Mice were sacrificed using CO2 euthanasia chamber and muscles were sampled at 8 h after last injection. All animal procedures were reviewed and approved by the Animal Research Committee of Tokushima University and Institutional Animal Care and Use Committee of RIKEN Kobe Branch.

**Table 1:** PCR and RT-qPCR primer sequences.

Gene name	Sense sequence (5′-3′)	Antisense sequence (5′-3′)	Accession no.	Purpose
*Fv2E*	AGTCATTAGGGGATGGGAGG	CCGTCTCCTGGGGAGATAGT	N/A	Genotyping
*Ddit3*	GCGACAGAGCCAGAATAACA	GATGCACTTCCTTCTGGAACA	NM_007837.4	qPCR
*Ppp1r15*	ACGATCGCTTTTGGCAAC	GACATGCTGGGGTCTTGG	NM_008654.2	qPCR
*Trib3*	CGCTTTGTCTTCAGCAACTGT	TCATCTGATCCAGTCATCACG	NM_175093.2	qPCR
*Ppargc1a*	CCGTAAATCTGCGGGATGATG	CAGTTTCGTTCGACCTGCGTAA	NM_008904.2	qPCR
*Fbxo32*	ACGACGTCGCAGCCAAGAAGAGAA	TCCATGGCGCTCCTTCGTACTTCC	NM_026346.3	qPCR
*Trim63*	CCTTCCTCTCAAGTGCCAAGCAGC	TGGCCCTCAAGGCCTCTGCTATGTG	NM_001039048.2	qPCR
*Gadd45*	CACTGTGTGCTGGTGACG	TTCCATTCGGATGCCATCAC	NM_007836.1	qPCR
*Rplp0*	TTTGGGCATCACCACGAAAA	GGACACCCTCCAGAAAGCGA	NM_007475.5	qPCR

### RT-qPCR analysis

Total RNA was extracted from skeletal muscle samples using AGPC methods, and was used as templates for cDNA synthesis with ReverTra Ace qPCR RT Master Mix and gDNA Remover (Toyobo). RT-qPCR reactions were performed using Prism 7900HT or Step One Plus Real-Time PCR Systems (Thermo Fisher) with AmpliTaq Gold® 360 Master Mix (Thermo Fisher), EvaGreen (Biotium), and the primers listed in Table 1. Ribosomal phosphoprotein P0 (Rplp0/36B4) mRNA was used as an internal control.

### Immunoblot analysis

Muscle lysates were prepared in RIPA buffer (50-mM Tris-HCl, 150-mM NaCl, 1% NP-40, 0.1% SDS, and 0.5% sodium deoxycholate) containing protease inhibitor cocktail (Nacalai Tesque) and phosphatase inhibitor cocktail (Biotool) using a Polytron homogenizer (Kinematica). The protein concentrations of lysates were measured using the bicinchoninic acid method, and immunoblot analyses were performed as described previously [12]. Proteins were detected using anti-PERK, anti-phospho-PERK (Thr980), anti-eIF2α, anti-phospho-eIF2α (Ser51), anti-Akt, anti-phospho-Akt (Ser473), anti-p70 S6 kinase (S6K), anti-phospho-S6K (Thr389), anti-eukaryotic translation initiation factor 4E (eIF4E)-binding protein (4EBP) 1, and anti-phospho-4EBP1 (Thr37/46), which were purchased from Cell Signaling Technology. GAPDH, which was purchased from MBL international, was used as a loading control. Bands were detected using WesternSure ECL Substrate (Li-Cor Biosciences), and images were acquired using an EZ-Capture II Cooled CCD Camera System (ATTO Corp.). The band intensity was determined using ImageJ software.

### Histological analyses

Skeletal muscle tissues were excised from euthanized mice, and were snap-frozen in a dry ice–acetone bath. Cryostat sections were stained with hematoxylin and eosin (HE). The relative cross-sectional area (CSA) was determined using ImageJ software from at least 200 muscle fibers of individual mice.

### Metabolomics analyses

Targeted profiling of amino acid metabolites was analyzed by CE–TOF/MS using an Agilent 7100 CE Capillary Electrophoresis system equipped with an Agilent 6230 Time-of-Flight mass spectrometer (Agilent Technologies) according to the methods described previously [13]. In brief, frozen skeletal muscle samples were homogenized with methanol containing internal standards (H3304-1002, Human Metabolome Technology Inc.); subsequently, both chloroform and Milli-Q water were added in the sample solution. The solution was centrifuged, and the aqueous fraction was centrifugally filtered through Ultrafree MC-PLHCC filter (Human Metabolome Technology Inc.). The filtrate was dried and dissolved in Milli-Q water containing reference compounds (H3304-1004, Human Metabolome Technology Inc.). CE-TOFMS conditions were as those reported previously [13]. Cationic metabolites were analyzed using a fused silica capillary (50μm i.d. × 80-cm total length) with Cation Buffer Solution (Human Metabolome Technologies) as the electrolyte. The sample was injected at a pressure of 50 mbar for 10s. The applied voltage was set at 27kV. Electrospray ionization-mass spectrometry was conducted in the positive ion mode, and the capillary voltage was set at 4,000V. The spectrometer was scanned from 50 to 1,000 m/z. An automatic recalibration of each acquired spectrum was performed using the masses of reference standards. For CE-MS system control and data acquisition, we used an Agilent MassHunter software for TOFMS (Agilent Technologies). All target metabolites were identified by matching their m/z values and migration times with the normalized m/z values and migration times of corresponding authentic standard compounds.

### Statistical analysis

Comparisons, except for data on body weight, were made using one-way ANOVA followed by Tukey–Kramer post-hoc tests. The hierarchical cluster analysis was performed using the statistical software R. Data were expressed as means ± standard errors of the mean (SEM), and differences were considered significant for *P* < 0.05, unless otherwise stated. Comparisons of body weights were performed using repeated-measures two-way analysis of variance.

## Results

### HSA-Fv2E-PERK Tg mice showed AP-dependent activation of the PERK pathway in skeletal muscles

**Fig 1:**
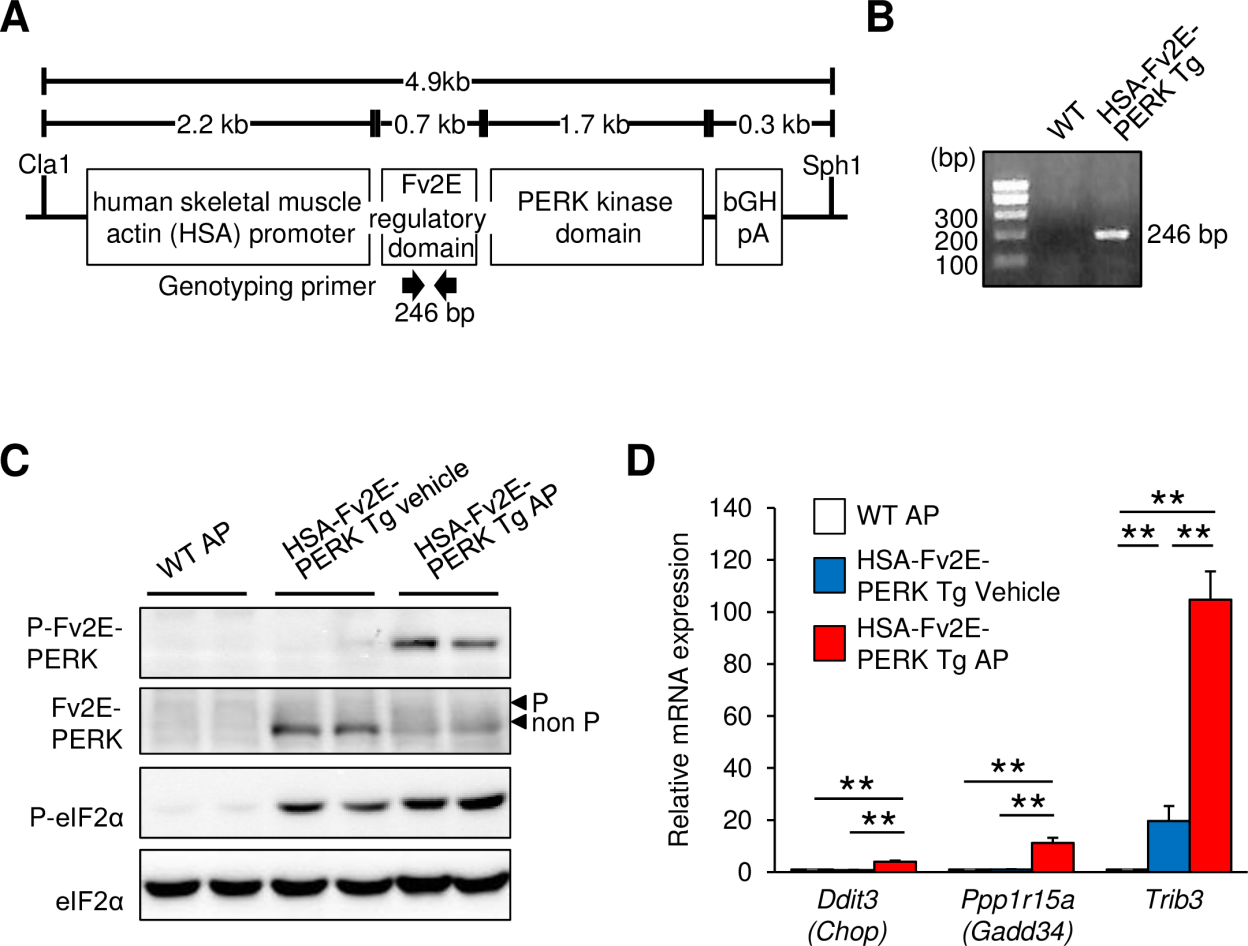
Structure of the transgene and PERK activation in skeletal muscles of HSA-Fv2E-PERK Tg mice. (A) Schema of the transgene for skeletal muscle-specific expression of Fv2E-PERK. (B) Representative PCR genotyping results from wild-type (WT) and HSA-Fv2E-PERK Tg mice. (C) Representative immunoblots of phosphorylated Fv2E-PERK (P), Fv2E-PERK (non P), phosphorylated eIF2α (P), and total eIF2α (non P) in the gastrocnemius muscles of WT and HSA-Fv2E-PERK Tg mice 8 h after intraperitoneal injections of vehicle or the artificial ligand AP21087 (AP) at 0.1 mg/kg body weight (BW). (D) RT-qPCR analyses of the expression of mRNA encoding targets of eIF2α phosphorylation in HSA-Fv2E-PERK Tg and WT mice at 8 h after administration of vehicle or AP (0.1 mg/kg BW). Data are presented as means ± standard errors of the mean (SEM; n = 3–5), and differences among AP-treated HSA-Fv2E-PERK Tg mice, vehicle-treated HSA-Fv2E-PERK Tg mice, and AP-treated WT mice were considered significant at *p < 0.05 or **p < 0.01.

To determine whether protein synthesis suppression inhibits the maintenance of skeletal muscle and causes muscle atrophy, we characterized the previously established Tg mouse model under the control of a human skeletal muscle actin (HSA) promoter that induces muscle-specific gene expression. The Tg mouse model expressed a chimeric protein comprising an Fv2E regulatory domain, which undergoes ligand-induced dimerization with the artificial ligand AP20187 (AP) and its PERK kinase domain, which is activated by dimerization (Fig 1A). The HSA-Fv2E-PERK Tg mice were genotyped and discriminated from wild-type mice using PCR primers for the artificial Fv2E regulatory domain (Fig 1B). eIF2α phosphorylation was very weak in steady-state skeletal muscles of wild-type mice injected with AP. However, because Fv2E-PERK was strongly expressed under the control of the HSA promoter in HSA-Fv2E-PERK Tg mice, eIF2α phosphorylation was observed even at a steady state (Fig 1C). The subsequent intraperitoneal injections of AP induced a phosphorylation-dependent mobility shift of Fv2E-PERK and corresponding Fv2E-PERK and eIF2α phosphorylation (Fig 1C). Wild-type and HSA-Fv2E-PERK Tg mice were then compared in mRNA expression analyses of genes that are induced by the PERK pathway. These RT-qPCR analyses showed that following AP injections, the expression of *Ddit3* (*Chop*), *Ppp1r15a* (*Gadd34*), and *Trib3* mRNAs increased in HSA-Fv2E-PERK Tg mice by 4-, 11-, and 105-fold, respectively, from that in AP-injected wild-type mice, which had a normal activation of the PERK pathway (Fig 1D).

### HSA-Fv2E-PERK Tg mice rapidly develop skeletal muscle atrophy following injections of AP

**Fig 2:**
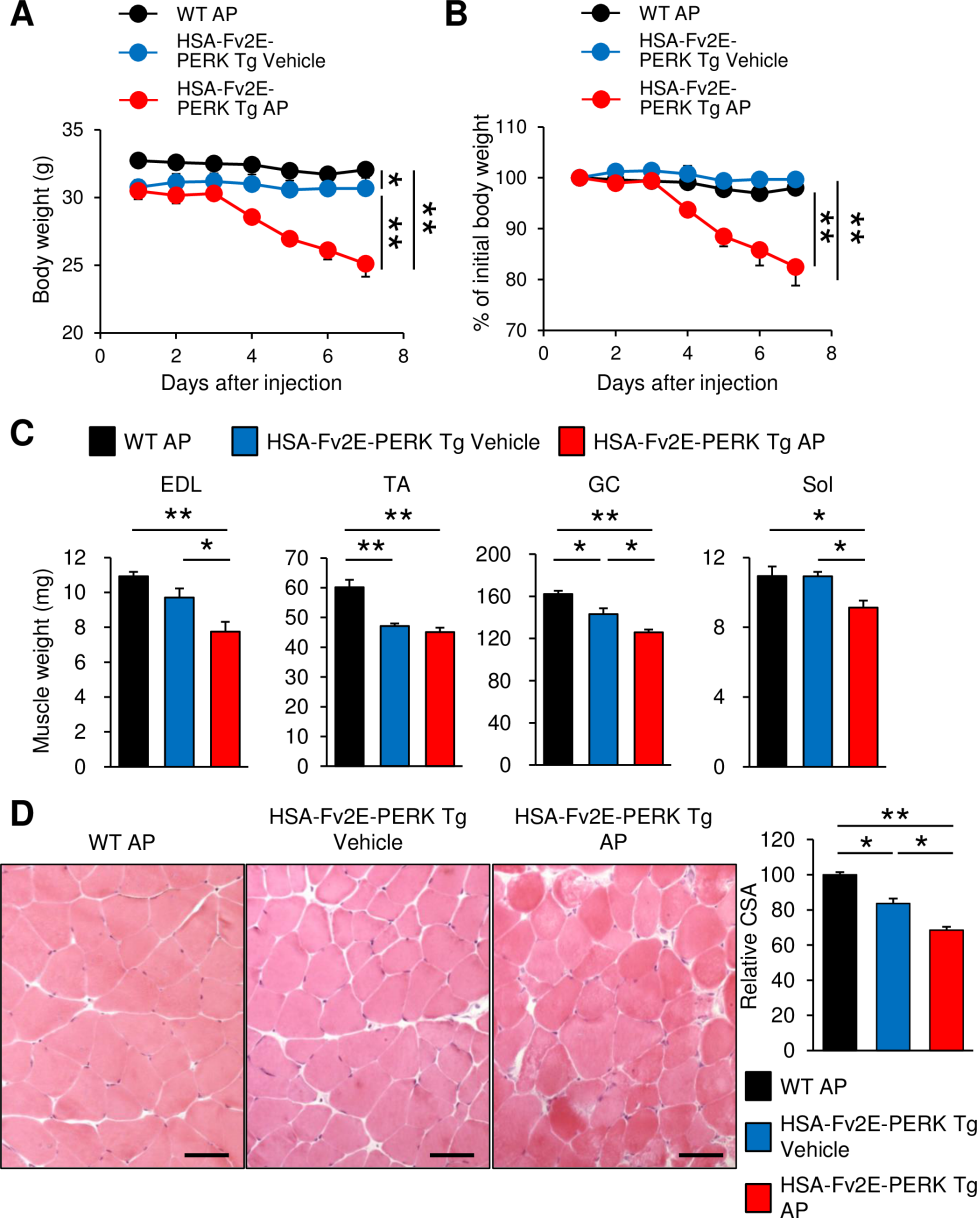
Acute skeletal muscle atrophy in AP-injected HSA-Fv2E-PERK Tg mice. (A) Body weight (BW) curves of AP-injected wild-type (WT) male, vehicle-injected HSA-Fv2E-PERK Tg mice, and AP-injected HSA-Fv2E-PERK Tg mice; vehicle or AP (0.1 mg/kg BW) were injected intraperitoneally once a day for 7 days (n = 5). (B) BW changes are expressed as percentages of initial BWs (Fig 2A). (C) Muscle weights of the extensor digitorum longus (EDL), tibialis anterior (TA), gastrocnemius (GC), and soleus (Sol) muscles of AP-injected WT, vehicle-injected Tg and AP-injected Tg mice; vehicle or AP (0.1 mg/kg BW) were intraperitoneally injected once a day for 7 days (n = 5). (D) Representative hematoxylin and eosin (H&E) staining of TA muscles of WT and HSA-Fv2E-PERK Tg mice. Vehicle or AP (0.1 mg/kg BW) was intraperitoneally injected once a day for 7 days. Right graph shows relative muscle cross-sectional area (CSA) of each genotype. Differences among AP-treated HSA-Fv2E-PERK Tg mice, vehicle-treated HSA-Fv2E-PERK Tg mice, and AP-treated WT mice treated were considered significant at *p < 0.05 or **p < 0.01.

At 6 months of age, the body weights (BWs) of the HSA-Fv2E-PERK Tg mice were slightly lower than those of the wild-type mice. However, following daily intraperitoneal injections of AP at 0.1 mg/kg BW, HSA-Fv2E-PERK Tg mice rapidly lost weight from the fourth day onwards (Fig 2A). Subsequently, BWs of the HSA-Fv2E-PERK Tg mice decreased by 17.5% from 30.5 ± 0.6 to 25.1 ± 1.0 g in 7 days (Fig 2A), corresponding with a 17% BW loss, whereas the wild-type mice showed almost no changes in BW (Fig 2B). To specifically investigate the loss of muscle weight following eIF2α phosphorylation, vehicle or AP (0.1 mg/kg BW) was intraperitoneally injected into wild-type and HSA-Fv2E-PERK Tg mice daily for 7 days, and hind limb muscle weights were measured (Fig 2C). In the AP-injected HSA-Fv2E-PERK Tg mice, weights of extensor digitorum longus (EDL), tibialis anterior (TA), gastrocnemius (GC), and soleus (Sol) muscles were reduced by 29%, 24%, 18%, and 13%, respectively, compared with those of AP-injected wild-type mice (Fig 2C). Accordingly, HE staining showed significantly higher frequencies of small fibers and degenerating myofibers in the AP-injected HSA-Fv2E-PERK Tg mice than in the AP-injected wild-type mice or vehicle-injected HSA-Fv2E-PERK Tg mice (Fig 2D). Muscle fiber CSA of TA muscles was reduced by 68% following AP injections in HSA-Fv2E-PERK Tg mice, compared with those of AP-injected wild-type mice (Fig 2D).

### Compensatory protein synthesis and degradation signaling but low mitochondrial function in muscles of AP-injected HSA-Fv2E-PERK Tg mice

**Fig 3:**
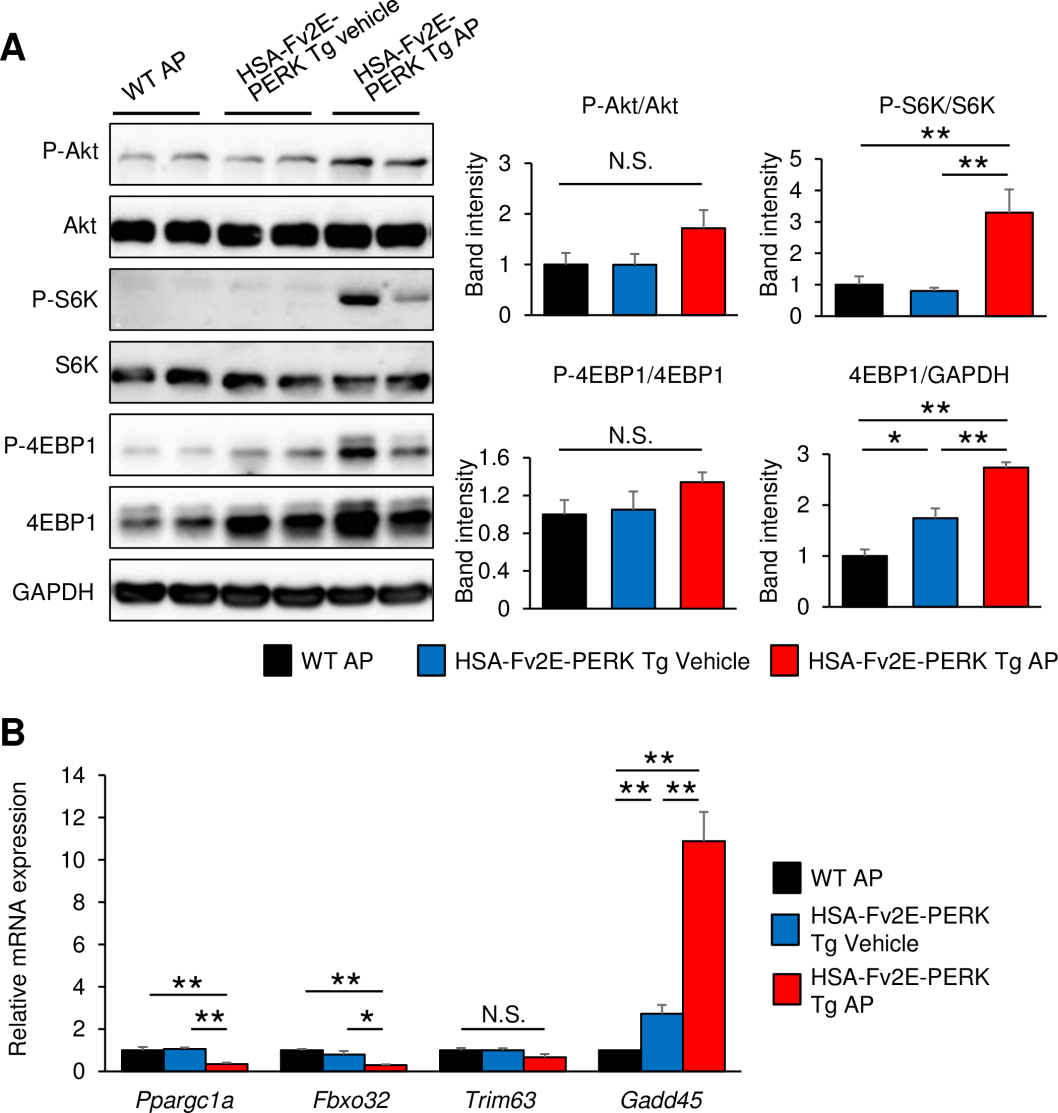
Modulation of intercellular signaling pathways that regulate skeletal muscle function in AP-injected HSA-Fv2E-PERK Tg mice. (A) Representative immunoblots of phosphorylated Akt, total Akt, phosphorylated S6K, total S6K, phosphorylated 4EBP-1, total 4EBP-1, and GAPDH at 8 h after vehicle or AP injections in gastrocnemius muscles of wild-type (WT) and HSA-Fv2E-PERK Tg mice; vehicle or AP (0.1-mg/kg BW) were intraperitoneally injected once a day for 7 days. Right graphs show the band intensity from images (n = 4–5). (B) RT-qPCR analyses of mRNAs encoding regulators of skeletal muscle function at 8 h after vehicle or AP injection in the gastrocnemius muscles of WT and HSA-Fv2E-PERK Tg mice; vehicle or AP (0.1 mg/kg BW) were intraperitoneally injected once a day for 7 days (n = 5). Differences among AP-treated HSA-Fv2E-PERK Tg mice, vehicle-treated HSA-Fv2E-PERK Tg mice, and AP-treated WT mice were considered significant at *p < 0.05 or **p < 0.01.

Hormones such as insulin and insulin-like growth factor 1 (IGF-1) increase muscle mass by accelerating protein synthesis and suppressing protein degradation. Moreover, Akt signaling is initiated by known protein synthesis hormone receptors that regulate both mTORC1 signaling and eIF2B activity, initiating protein synthesis [14]. Therefore, we investigated changes in the Akt and following mTORC1 signaling in AP-injected HSA-Fv2E-PERK Tg mice using immunoblotting experiments. Following seven daily AP injections, HSA-Fv2E-PERK Tg mice did not show increased phospho-Akt/total Akt ratio but showed increased levels of phosphorylated protein/total protein ratio of the mTORC1 signaling targets, such as S6K and 4EBP1 (Fig 3A). Accordingly, eIF4E initiates translation by binding to mRNA cap structures and recruiting ribosomes, whereas 4EBP1 suppresses translation by binding to eIF4E and thus inhibits eIF4E activity. Conversely, highly phosphorylated 4EBP1 dissociates from eIF4E and activates translation. In addition, phosphorylated S6K accelerates binding of mRNA to 40S ribosomal subunits and promotes protein synthesis. Taken together with the present observations, these data suggest that mTORC1 signaling for the formation of the 48S preinitiation complex are activated in AP-injected HSA-Fv2E-PERK Tg mice.

GADD45a transcription is induced by FoxO and ATF4 [15, 16] and is reportedly induced by various stresses that cause muscle atrophy [16, 17]. Because GADD45a is considered to be a mediator of muscle atrophy [16], we investigated GADD45a mRNA expression in AP-injected HSA-Fv2E-PERK Tg mice. These experiments showed 10-fold greater GADD45a expression in HSA-Fv2E-PERK Tg mice after seven daily intraperitoneal AP injections (one per day) compared with that in AP-injected wild-type mice (Fig 3B). Previous studies have shown that the Akt pathway suppresses protein degradation by inactivating FoxO, which induces Fbxo32 (MAFbx) [18, 19] and Trim63 (MuRF1) [20]. These proteins are E3 ubiquitin-ligases involved in protein degradation in skeletal muscles. Thus, we analyzed changes in the levels of Fbxo32 and Trim63 mRNA in AP-injected HSA-Fv2E-PERK Tg mice. Following 7 days of intraperitoneal injections of AP (once per day) into HSA-Fv2E-PERK Tg mice, Fbxo32 mRNA levels were significantly decreased to approximately 25% of those in wild-type mice (Fig 3B). These results indicate Fbxo32 suppression in AP-injected HSA-Fv2E-PERK Tg mice, suggesting negative regulation of protein degradation.

PPARGC1A (PGC1α) regulates mitochondria biogenesis and is important for skeletal muscle functions. Accordingly, PGC1α-knockout mice show the accumulation of abnormal mitochondria and reduced muscle mass and strength [21, 22]. Thus, we investigated PPARGC1A mRNA expression in AP-injected HSA-Fv2E-PERK Tg mice using RT-qPCR. In these experiments, PPARGC1A expression decreased in AP-injected HSA-Fv2E-PERK Tg mice to approximately 25% of that in AP-injected wild-type mice after 7 days (Fig 3B), suggesting negative regulation of mitochondrial function.

These results indicate pronounced muscle atrophy and suggest that compensatory activation of the mTORC1 signaling leads to increased protein synthesis and suppression of protein degradation in the muscles of AP-injected HSA-Fv2E-PERK Tg mice. In contrast, expression of the mitochondrial regulator PPARGC1A was low in the muscles of these mice, suggesting that eIF2α phosphorylation and signaling might be coupled with mitochondrial biogenesis.

### Impaired intracellular amino-acid homeostasis in muscles of AP-injected HSA-Fv2E-PERK Tg mice

**Fig 4:**
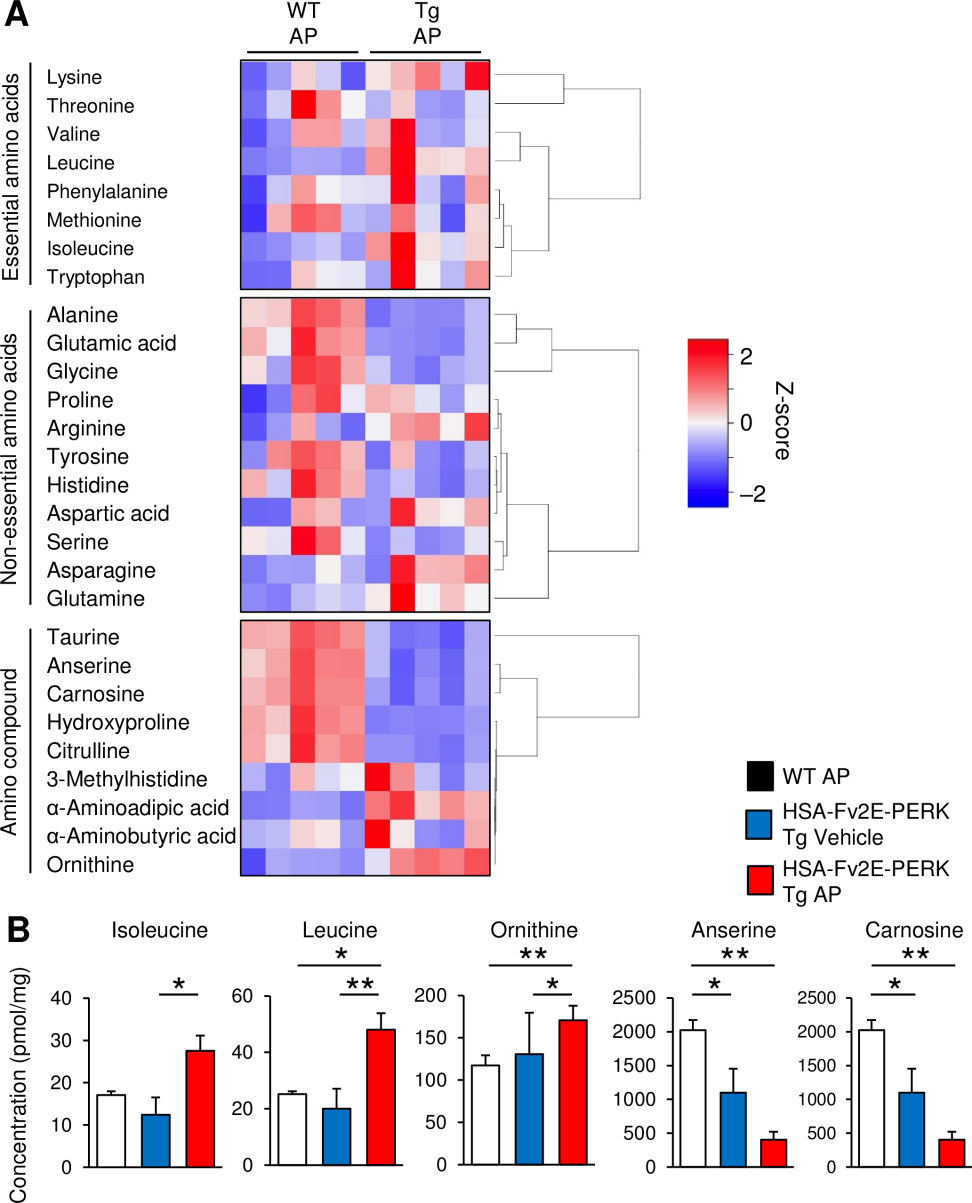
Altered intercellular amino acid metabolism in AP-injected HSA-Fv2E-PERK Tg mice. (A) Heatmap representation of amino acid metabolites in gastrocnemius muscles of AP-injected WT and AP-injected Tg mice (n = 5). Colors indicate ratios of determined amino acids contents in vehicle-injected WT mice; the vertical axis is labeled with a continuous scale color bar key. AP (1-μg/kg BW) was injected intraperitoneally. (B) Amino acid concentrations in gastrocnemius muscles of AP-injected WT, vehicle-injected Tg, and AP-injected Tg mice. Vehicle or AP (0.1-mg/kg BW) was intraperitoneally injected once a day for 7 days (n = 5). Differences among AP-treated HSA-Fv2E-PERK Tg mice, vehicle-treated HSA-Fv2E-PERK Tg mice, and AP-treated WT mice were considered significant at *p < 0.05 or **p < 0.01.

Protein synthesis suppression in skeletal muscles may lead to increased intracellular amino acid levels, reflecting decreased utilization in protein synthesis. Moreover, increases in the free amino acid levels in muscle cells stimulate the synthesis of muscle proteins and protein anabolism [23], in which essential amino acids play central roles [24]. In particular, the branched chain amino acid leucine reportedly activates the mTOR signaling pathway and stimulates 4EBP1 and S6K expression and protein anabolism [25, 26]. However, eIF2α phosphorylation induces the translation of ATF4 and transcription of genes involved in amino acid metabolism, such as amino acid transporters in mouse fibroblast and liver cells [27, 28]. In a recent study, we found that genes, which are involved in serine metabolism, were induced by eIF2α phosphorylation in skeletal muscles [11]. These studies suggest that intracellular free amino acid levels are closely linked to the performance of skeletal muscles. Thus, in further experiments, we compared the intracellular amino acid profiles of 35 intracellular amino acids and related amino compounds in skeletal muscles from AP-injected HSA-Fv2E-PERK Tg mice and wild-type mice using liquid chromatography tandem mass spectrometry analyses. Among 20 amino acids, cysteine was excluded because it is easily oxidized to cystine during analysis. Subsequently, we divided amino acid metabolites into three groups: essential amino acid group, non-essential amino acid group, and amino compound group. Hierarchical cluster analyses were then employed to uncover a multitude of amino acid relationships among each group. According to the amino acid cluster formations, amino compound group was markedly different between AP-injected HSA-Fv2E-PERK Tg mice and wild-type mice (Fig 4A). In AP-injected HSA-Fv2E-PERK Tg mice, increases in the levels of the branched chain amino acid leucine and isoleucine indicate the activation of mTORC1 signaling (Fig 4B). AP-injected HSA-Fv2E-PERK Tg mice had more ornithine, lysine, and lysine metabolite, α-aminoadipic acid, but less ornithine metabolites, hydroxyproline and citrulline, than AP-injected wild-type mice. On the other hand, these HSA-Fv2E-PERK Tg mice had lower levels of histidine, its metabolites, carnosine, and anserine (Fig 4B). Furthermore, the muscle-abundant amino acids alanine, glutamate, and taurine were present at decreased concentrations in the HSA-Fv2E-PERK Tg mice compared with those in the wild-type mice, which was in agreement with their reduced muscle mass. These data indicate that HSA-Fv2E-PERK Tg mice accumulate intracellular free amino acids, with characteristic profiles of specific amino acid levels.

## Discussion

Muscle mass loss directly contributes to intolerance to exercise and impaired daily activities, which makes it a strong determinant of quality of life and mortality [29–31]. At present, no therapeutic interventions are established to successfully treat muscle wasting. Therefore, the development of model of muscle wasting is expected for exploring and validating the new therapeutic candidates. Here, we characterized the change in the skeletal muscles of HSA-Fv2E-PERK Tg mice treated with AP for successive 7 days. Strong and continuous eIF2α phosphorylation induced by AP treatment led to rapid body weight loss due to drastically reduced muscle mass in the AP-injected HSA-Fv2E-PERK Tg mice. A metabolomic analysis of intracellular free amino acid in skeletal muscles identified the characteristic profile associated with reduced 43S preinitiation complex.

**Fig 5:**
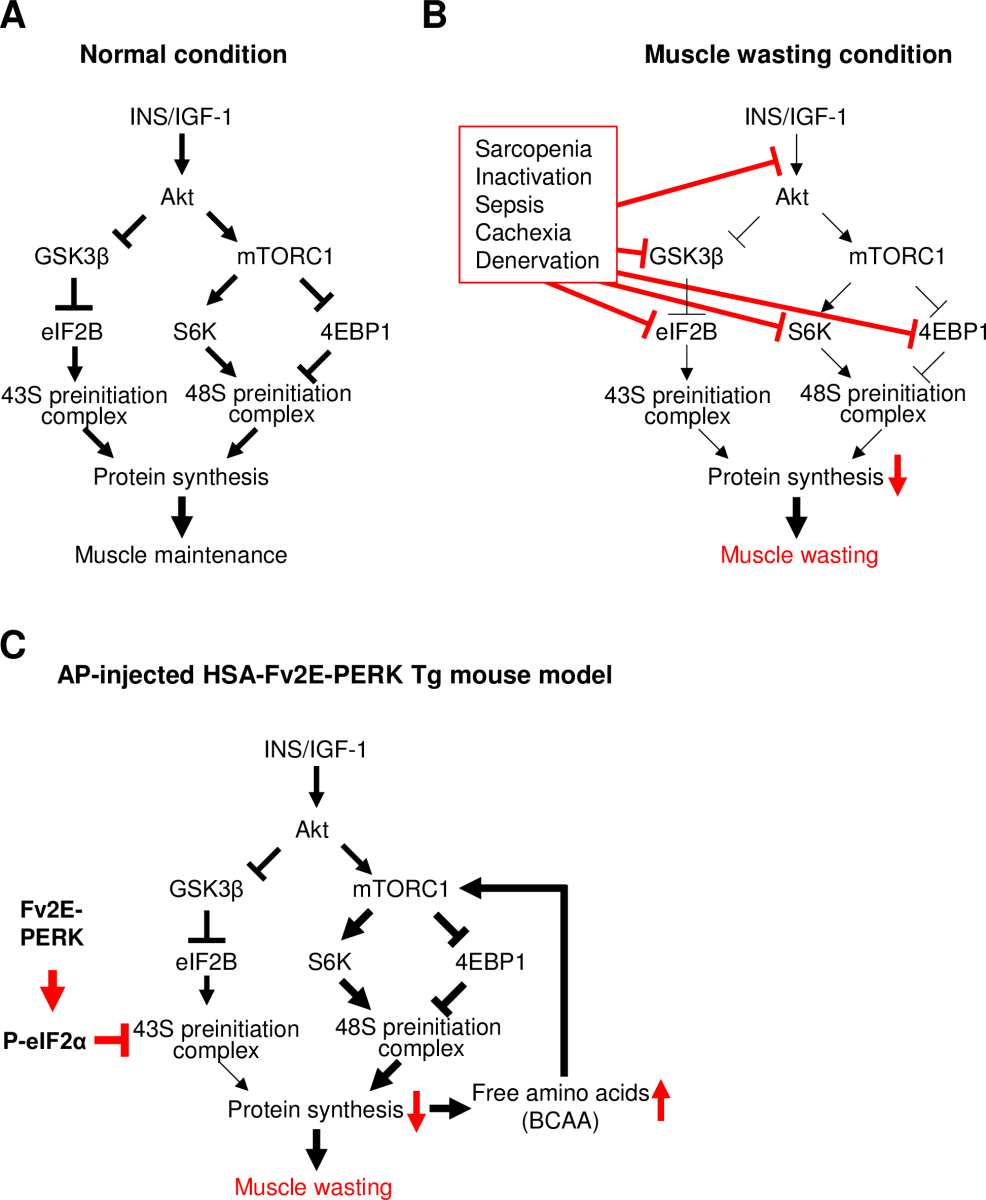
Schematic representation of muscle wasting in AP-injected HSA-Fv2E-PERK Tg mouse. (A) In normal condition, hormones, such as insulin and IGF-1, stimulate protein synthesis through enhancing the formation of the 43S and 48S preinitiation complexes, which are essential for muscle maintenance. (B) In muscle wasting conditions, signals regulating the formation of the 43S and 48S preinitiation complex were disturbed. (C) In AP-injected HSA-Fv2E-PERK Tg mouse, inhibition of the formation of 43S preinitiation complex by eIF2 phosphorylation led to muscle atrophy due to reduced protein synthesis, along with a counter-regulatory increase in 48S preinitiation complex.

Accumulating evidence suggest that multiple concurrent processes in protein synthesis are disturbed during muscle wasting conditions (Fig 5). In normal condition, hormones, such as insulin and IGF-1, control skeletal muscle growth thorough Akt phosphorylation. Akt activation leads to both mTORC1 activation and glycogen synthase kinase 3β (GSK-3β) inactivation, leading to eIF2B GEF activity inhibition [32]. In muscle wasting condition due to disuse atrophy, reduced Akt1 and Akt2 expression as well as Akt phosphorylation occur [33]. Reduced signaling through mTORC1 including decreased 4EBP1 and S6K1 phosphorylation was observed in sarcopenia [34], sepsis-induced muscle atrophy [35], and cancer cachexia [36]. GSK-3 phosphorylation and eIF2B subunit expression were reduced in sepsis-induced muscle atrophy [35] and sarcopenia [34]. In fact, a skeletal muscle-specific IGF1 receptor knockout mouse model was previously developed, which showed reduced muscle fiber numbers and sizes [37]. Moreover, a skeletal muscle-specific mTORC1 knockout mouse model was shown to manifest skeletal muscle atrophy at 6–9 weeks of age [38, 39]. These mice carrying genetic modifications of signals related to protein synthesis can serve as muscle wasting models. To the best of our knowledge, there is no muscle wasting mouse model caused by repressed 43S preinitiation complex assembly, and the HSA-Fv2E-PERK Tg mouse is the first such model. Predominant contribution of repressed 43S preinitiation complex assembly compared with that of 48S preinitiation complex assembly in protein synthesis has been suggested *in vitro* using cultured cells [40] but has not been addressed *in vivo*. The AP-injected HSA-Fv2E-PERK Tg mice led to reduced muscle mass despite the counter regulatory activation of mTORC1 signaling, indicating, for the first time, that the inhibition of 43S preinitiation complex assembly is dominant to that of 48S preinitiation complex assembly in protein synthesis *in vivo*. In addition, HSA-Fv2E-PERK Tg mice develop more severe and rapid skeletal muscle atrophy than any of the previous sarcopenia mouse models, offering a superior tool for further studies.

PERK is activated by endoplasmic reticulum (ER) stress. Several reports demonstrate that ER stress and unfolded protein response (UPR) pathway get activated under various muscle conditions such exercise [41], aging[42], cancer cachexia[43], sepsis[44] and myopathy[45]. ER stress-induced UPR suggests to play an important role in maintaining skeletal muscle mass and function. For example, deletion of the ATF6 branch of the UPR pathway compromised efficient muscle recovery from acute exercise in mouse [41]. Inhibition of ER stress by a chemical chaperon caused skeletal muscle wasting in cancer cachexia model mice[43]. However, prolonged activation of the UPR pathway and unresolved ER stress can lead to many pathological processes in different tissues. Indeed, our results indicate that persistent activation of PERK signaling reduces skeletal muscle mass. Therefore, mild and transit UPR activation would be beneficial to the muscle but strong and continuous UPR activation would be deleterious. Because the exact roles of different arms of the UPR remain enigmatic, further investigation is needed to determine.

Inhibited protein synthesis appeared to increase the intracellular free amino acid levels, but interestingly, not all amino acid levels increased, and some amino acid levels decreased, compared with the AP-injected wild-type mice. Increased levels of branched chain amino acids, such as leucine and isoleucine, are responsible for mTORC1 signaling activation. On the other hands, imidazole dipeptides, such as carnosine and anserine, which are known to function as natural antioxidants required for adaptation to anaerobic exercise, were decreased. These altered intracellular free amino acid profiles might serve as biomarkers for reduced protein synthesis caused by repressed 43S preinitiation complex assembly. It is interesting to note the mechanism underlying these alternations. Other authors and we have previously reported that eIF2α phosphorylation regulates amino acid metabolism by inducing the amino acid transporter proteins through transcriptional induction via ATF4. However, the changes in intracellular free amino acids could be due to a mixed profile of inhibited eIF2B activity, increased mTORC1 signaling, and other compensation mechanisms. Therefore, the altered profile could not be directly attributed to the change in amino acid transport mediated by eIF2α phosphorylation. Further detailed analysis will be required to reveal the cooperative mechanism. Hence, HSA-Fv2E-PERK Tg mice will facilitate future investigations of disease mechanisms and the development of novel drugs for the treatment of muscle wasting.
